# Effects of *MTNR1B* genetic variants on the risk of type 2 diabetes mellitus: A meta‐analysis

**DOI:** 10.1002/mgg3.611

**Published:** 2019-02-27

**Authors:** Ling‐long Shen, Yin Jin

**Affiliations:** ^1^ Department of Clinical Laboratory Huzhou Maternity and Child Health Care Hospital Huzhou China; ^2^ Department of Clinical Laboratory Huzhou Central Hospital Huzhou China

**Keywords:** genetic variants, melatonin receptor 1B (*MTNR1B*), meta‐analysis, type 2 diabetes mellitus (T2DM)

## Abstract

**Background:**

Whether melatonin receptor 1B (*MTNR1B*) variants are associated with type 2 diabetes mellitus (T2DM) remains unclear. Therefore, we performed this meta‐analysis to better explore correlations between *MTNR1B* variants and T2DM.

**Methods:**

Literature research was performed in PubMed, Medline, and Embase. Odds ratios (ORs) and 95% confidence intervals (CIs) were calculated.

**Results:**

Totally 21 studies were enrolled to analyses. Pooled overall analyses showed that *MTNR1B* rs10830963 variant was significantly correlated with the susceptibility to T2DM (allele model: *p* = 0.02, OR = 0.97, 95% CI 0.95–1.00). Further subgroup analyses by ethnicity of participants revealed that rs10830963 variant was significantly correlated with the susceptibility to T2DM in South Asians, but not in Caucasians or East Asians. No any other positive results were found in overall and subgroup analyses.

**Conclusions:**

Our findings indicated that *MTNR1B* rs10830963 variant might serve as a genetic biomarker of T2DM, especially in South Asians.

## 1 INTRODUCTION

Type 2 diabetes mellitus (T2DM), characterized by chronic hyperglycemia resulted from resistance against insulin, is the most prevalent metabolic disorder worldwide, and it currently affects over 300 million people all over the world (American Diabetes Association, 2014; Zheng, Ley, & Hu, 2018). To date, the exact underlying pathogenic mechanism of T2DM is still unclear. Nevertheless, accumulating evidence support that genetic predisposition factors may play a crucial part in its pathogenesis. First, it was proved that positive family history is a strong independent risk factor of T2DM (Papazafiropoulou, Papanas, Melidonis, & Maltezos, 2017). Second, over one hundred genetic loci were found to be correlated with an increased risk of T2DM by past genome‐wide association studies (Gaulton, 2017). Overall, these findings jointly supported that genetic factors are crucial for the occurrence and development of T2DM.

Melatonin—a pineal gland hormone that is responsible for regulating circadian rhythm— can also impact glucose metabolism by affecting circadian (Claustrat & Leston, 2015). Previous experimental studies found that melatonin receptor (MTNR) was abundantly expressed in pancreatic islet, and plasma melatonin level was reversely correlated with insulin level (Espino, Pariente, & Rodríguez, 2011; Lardone, Alvarez‐Sanchez, Guerrero, & Carrillo‐Vico, 2014; Singh & Jadhav, 2014). Consequently, it is rational to believe that genetic variants of *MTNR* might influence melatonin function and impact individual susceptibility to T2DM.

So far, several studies already investigated potential roles of *MTNR1B* in T2DM. But the results of these studies were inconsistent (Hu & Jia, 2016; She, Laudon, & Yin, 2014). Therefore, we performed the present meta‐analysis to better evaluate potential associations between *MTNR1B* genetic variants and T2DM.

## 2 MATERIALS AND METHODS

### 2.1 Literature search and inclusion criteria

This meta‐analysis adhered to the Preferred Reporting Items for Systematic Reviews and Meta‐analyses (PRISMA) guideline (Moher, Liberati, Tetzlaff, & Altman; PRISMA Group, 2009). Potentially relevant literatures that were published before November 2018 were retrieved from PubMed, Medline, and Embase using the following searching strategy: (melatonin receptor type 1B OR MTNR1B) AND (polymorphism OR variant OR mutation OR genotype OR allele) AND (type 2 diabetes mellitus OR T2DM). We also screened the references of retrieved articles to identify other potentially relevant studies.

To test the research hypothesis of this meta‐analysis, included studies must meet all the following criteria: (a) case–control study on correlations between *MTNR1B* genetic variants and T2DM; (b) provide genotypic and/or allelic frequency of investigated variants in cases and controls; (c) full text in English available. Studies were excluded if one of the following criteria was fulfilled: (a) not relevant to *MTNR1B* genetic variants and T2DM; (b) case reports or case series; (c) abstracts, reviews, comments, letters, and conference presentations. For duplicate publications, we only included the study with the largest sample size for analyses.

### 2.2 Data extraction and quality assessment

The following data were extracted from included studies: (a) name of the first author; (b) publication time; (c) country and ethnicity; (d) sample size; and (e) genotypic distributions of *MTNR1B* variants in cases and controls. The probability value (*P‐*value) of Hardy‐Weinberg equilibrium (HWE) was also calculated. When necessary, we wrote to the corresponding authors for raw data. We used the Newcastle–Ottawa scale (NOS) to assess the quality of eligible studies (Stang, 2010). This scale has a score range of 0 to 9, and studies with a score of more than seven were thought to be of high quality. Two experienced reviewers conducted data extraction and quality assessment independently. Any disagreement between two reviewers was solved by discussion until a consensus was reached.

### 2.3 Statistical analyses

All statistical analyses were conducted with Review Manager Version 5.3.3 (The Cochrane Collaboration, Software Update, Oxford, United Kingdom). Odds ratios (ORs) and 95% confidence intervals (CIs) were calculated to estimate strength of associations between *MTNR1B* and T2DM in all possible genetic models, and *P‐*values ≤0.05 were considered to be statistically significant. Between‐study heterogeneities were evaluated by *I* ^2^ statistic. If *I* ^2^ was greater than 50%, random effect models (REMs) would be used to pool the data. Otherwise, fixed effect models (FEMs) would be employed for synthetic analyses. Subgroup analyses by ethnicity of participants were subsequently performed. Sensitivity analyses were conducted to examine the stability of synthetic results. Funnel plots were used to evaluate possible publication biases.

## 3 RESULTS

### 3.1 Characteristics of included studies

**Figure 1:**
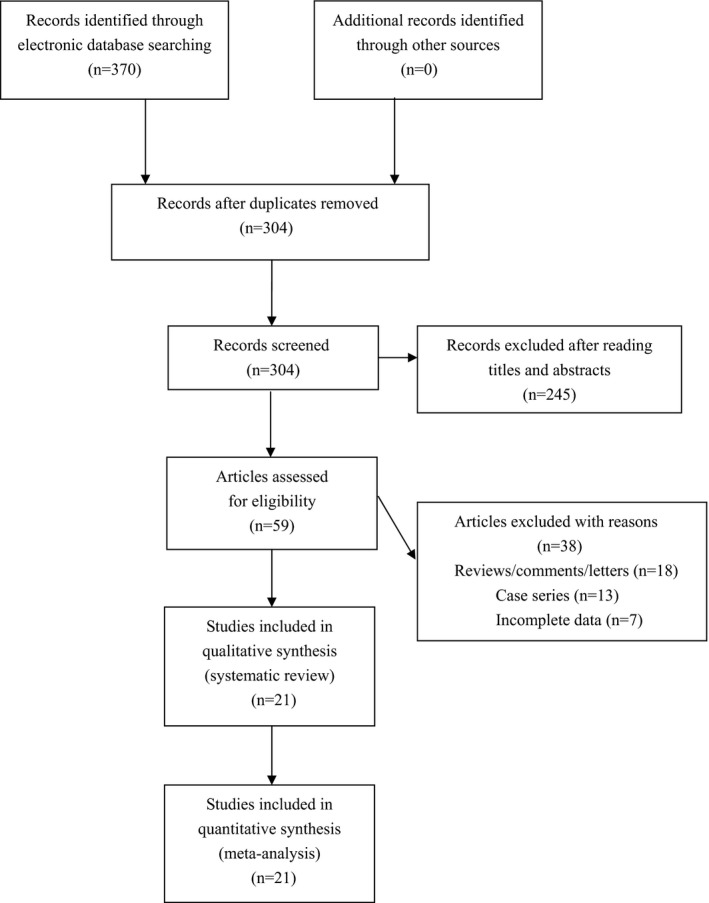
Flowchart of study selection for the present study

We found 370 potential relevant articles. Among these articles, a total of 21 eligible studies were finally included for synthetic analyses (see Figure 1). The NOS score of eligible articles ranged from 7 to 8, which indicated that all included studies were of high quality. Baseline characteristics of included studies were shown in Table 1.

**Table 1:** The characteristics of included studies for *MTNR1B* genetic variants and T2DM

First author, year	Country	Ethnicity	Type of disease	Sample size	Genotype distribution		*P*‐Value for HWE	NOS score
					Cases	Controls		
rs1387153					CC/CT/TT			
Bai, 2015	China	East Asian	T2DM	497/469	NA	NA	NA	7
Been, 2012	USA	Mixed	T2DM	1,164/973	459/558/147	363/479/131	0.171	8
Huerta‐Chagoya, 2015	Mexico	Mixed	T2DM	4,366/3,848	NA	NA	NA	7
Kan, 2010	China	East Asian	T2DM	1,912/2,041	587/969/356	688/996/357	0.915	7
Ohshige, 2011	Japan	East Asian	T2DM	2,839/2,125	NA	NA	NA	7
Qian, 2015	China	East Asian	T2DM	1,180/1,186	NA	NA	NA	7
Salman, 2015	India	South Asian	T2DM	346/341	NA	NA	NA	7
Tabara, 2011	Japan	East Asian	T2DM	495/399	196/226/73	139/195/65	0.807	8
rs4753426					TT/TC/CC			
Dietrich, 2011	Germany	Caucasian	T2DM	100/820	28/47/25	208/439/173	0.037	8
Patel, 2018	India	South Asian	T2DM	426/481	123/201/102	134/252/95	0.230	7
rs10830962					GG/GC/CC			
Patel, 2018	India	South Asian	T2DM	417/470	114/205/98	122/226/122	0.406	7
Salman, 2015	India	South Asian	T2DM	346/341	NA	NA	NA	7
rs10830963					CC/CG/GG			
Been, 2012	USA	Mixed	T2DM	1,169/1,001	435/560/174	393/445/163	0.052	8
Dietrich, 2011	Germany	Caucasian	T2DM	103/821	56/44/3	439/327/55	0.573	8
Fujita, 2012	Japan	East Asian	T2DM	2,592/2017	NA	NA	NA	7
Gao, 2016	China	East Asian	T2DM	724/759	243/347/134	280/350/129	0.274	7
Hu, 2010	China	East Asian	T2DM	3,410/3,412	NA	NA	NA	7
Kan, 2010	China	East Asian	T2DM	1,912/2,041	585/960/367	675/989/350	0.707	7
Ling, 2011	China	East Asian	T2DM	1,118/1,161	403/538/177	404/590/167	0.039	8
Lyssenko, 2009	Sweden	Caucasian	T2DM	2,201/16,630	NA	NA	NA	7
Ohshige, 2011	Japan	East Asian	T2DM	2,839/2,125	NA	NA	NA	7
Patel, 2018	India	South Asian	T2DM	434/489	133/266/35	169/259/61	0.012	7
Rees, 2011	UK	Caucasian	T2DM	1,667/1,568	631/753/283	583/714/271	0.040	8
Reiling, 2009	The Netherlands	Caucasian	T2DM	2,537/1,990	1,343/1,011/183	1,111/764/115	0.275	8
Rönn, 2009	Sweden	Caucasian	T2DM	1,165/1,105	371/553/241	374/558/173	0.139	7
Salman, 2015	India	South Asian	T2DM	341/346	NA	NA	NA	7
Semiz, 2014	Bosnia and Herzegovina	Caucasian	T2DM	162/106	96/58/8	51/47/8	0.527	7
Sparsø, 2009	Denmark	Caucasian	T2DM	6,055/1,948	3,228/2,360/467	1,002/776/170	0.260	7
Tabara, 2011	Japan	East Asian	T2DM	488/398	181/230/77	134/192/72	0.824	8
Tam, 2010	China	East Asian	T2DM	1,342/1,644	448/633/261	523/789/332	0.273	8

Note

HWE: Hardy–Weinberg equilibrium; NOS: Newcastle–Ottawa scale; NA: not available.

HWE assumes that allele and genotype frequencies in a population will remain constant from generation to generation in the absence of other evolutionary influences. Consider a population of monoecious diploids, where each organism produces male and female gametes at equal frequency, and has two alleles at each gene locus. The allele frequencies at each generation are obtained by pooling together the alleles from each genotype of the same generation according to the expected contribution from the homozygote and heterozygote genotypes.

### 3.2 Overall and subgroup analyses

To investigate potential correlations between *MTNR1B* genetic variants and T2DM, eight studies about rs1387153 variant (12,799 cases and 11,382 controls), two studies about rs4753426 variant (526 cases and 1,301 controls), two studies about rs10830962 variant (763 cases and 811 controls), and eighteen studies about rs10830963 variant (30,259 cases and 39,561 controls) were enrolled to analyses. A significant association with the susceptibility to T2DM was detected for rs10830963 variant (allele model: *p* = 0.02, OR = 0.97, 95% CI 0.95–1.00) in overall analyses. Further subgroup analyses according to ethnicity of participants revealed that rs10830963 variant was significantly correlated with the susceptibility to T2DM in South Asians, but not in East Asians or Caucasians. No any other positive results were found in overall and subgroup analyses (see Table 2 and Supplementary Figure 1).

**Table 2:** Overall and subgroup analyses for *MTNR1B* genetic variants and T2DM

Polymorphisms	Population	Sample size (cases/controls)	Dominant comparison		Recessive comparison		Additive comparison		Allele comparison	
			*P‐*value	OR (95% CI)	*P‐*value	OR (95% CI)	*P* value	OR (95% CI)	*P* value	OR (95% CI)
rs1387153	Overall	12,799/11,382	0.73	0.97 (0.85–1.12)	0.79	1.02 (0.91–1.13)	0.92	1.00 (0.92–1.08)	0.48	0.98 (0.92–1.04)
	East Asian	6,923/6,220	0.93	0.99 (0.83–1.19)	0.57	1.04 (0.92–1.17)	0.83	1.01 (0.92–1.11)	0.22	0.95 (0.87–1.03)
rs4753426	Overall	526/1,301	0.56	0.93 (0.72–1.19)	0.08	1.27 (0.97–1.65)	0.05	0.80 (0.64–1.00)	0.51	0.95 (0.81–1.11)
rs10830962	Overall	763/811	0.40	0.88 (0.65–1.19)	0.64	1.07 (0.80–1.45)	0.75	1.04 (0.80–1.36)	0.31	0.97 (0.81–1.08)
	South Asian	763/811	0.40	0.88 (0.65–1.19)	0.64	1.07 (0.80–1.45)	0.75	1.04 (0.80–1.36)	0.31	0.97 (0.81–1.08)
rs10830963	Overall	30,259/39,561	0.44	0.98 (0.94–1.03)	0.81	1.01 (0.91–1.13)	0.84	1.00 (0.96–1.05)	**0.02**	**0.97 (0.95–1.00)**
	Caucasian	13,890/24,168	0.99	1.00 (0.90–1.11)	0.68	1.05 (0.84–1.30)	0.36	0.97 (0.91–1.03)	0.53	0.97 (0.89–1.06)
	East Asian	14,425/13,557	0.68	0.98 (0.91–1.06)	0.25	1.06 (0.96–1.16)	1.00	1.00 (0.93–1.08)	0.18	0.98 (0.94–1.01)
	South Asian	775/835	0.21	0.84 (0.63–1.10)	**0.03**	**0.62 (0.40–0.95)**	**0.01**	**1.41 (1.08–1.83)**	0.72	0.97 (0.85–1.13)

Note

OR: odds ratio; CI: confidence interval, NA: not available.

The values in bold represent there is statistically significant differences between cases and controls.

### 3.3 Sensitivity analyses

We performed sensitivity analyses by excluding studies that deviated from HWE. No alterations of results were detected in sensitivity analyses, which suggested that our findings were statistically reliable.

### 3.4 Publication biases

Publication biases were evaluated with funnel plots. We did not find obvious asymmetry of funnel plots in any comparisons, which indicated that our findings were unlikely to be impacted by severe publication biases.

## 4 DISCUSSION

To the best of our knowledge, this is so far the most comprehensive meta‐analysis on correlations between *MTNR1B* genetic variants and T2DM, and our pooled analyses demonstrated that rs10830963 variant may be correlated with susceptibility to T2DM, especially in South Asians.

There are several points that need to be addressed about this meta‐analysis. Firstly, previous experimental studies showed that mutant allele of rs10830963 variants was correlated with altered glucose level and B‐cell function, which may partially explain our positive finding (Li et al., 2018; Staiger et al., 2008). Secondly, the pathogenic mechanism of T2DM is highly complex, and hence it is unlikely that a single genetic variant could significantly contribute to its development. As a result, to better illustrate potential correlations of certain genetic variants with T2DM, we strongly recommend further studies to perform haplotype analyses and explore potential gene–gene interactions.

Like all meta‐analyses, this study certainly has some limitations. First, our results were derived from unadjusted analyses due to lack of raw data, and lack of further adjusted analyses for potential confounding factors may impact the reliability of our findings (Xie, Shi, & Liu, 2017; Xie, Shi, Xun, & Rao, 2017). Second, obvious heterogeneities were found in several subgroups, which indicated that the controversial results of included studies could not be fully explained by differences in ethnic background, and other baseline characteristics of participants may also contribute to between‐study heterogeneities (Shi, Xie, Jia, & Li, 2016). Third, associations between *MTNR1B* genetic variants and T2DM may also be modified by gene–gene and gene–environmental interactions. However, most eligible studies ignore these potential interactions, which impeded us to perform relevant analyses accordingly. To sum up, our findings should be cautiously interpreted on account of above mentioned limitations.

In conclusion, our meta‐analysis suggested that *MTNR1B* rs10830963 variant might serve as a genetic biomarker of T2DM, especially in South Asians. However, further well‐designed studies are still warranted to confirm our findings.

## ETHICAL APPROVAL

This article does not contain any studies with human participants or animals performed by any of the authors.

## CONFLICT OF INTEREST

The authors declare that they have no conflict of interest.

## AUTHORS' CONTRIBUTIONS

Ling‐long Shen and Yin Jin conceived the study, participated in its design, conducted the systematic literature review, performed data analyses, and drafted the manuscript. All the authors gave final approval and agreed to be accountable for all aspects of work ensuring integrity and accuracy.
