# Supplementary material for: Effects of MTNR1B genetic variants on the risk of type 2 diabetes mellitus: A meta‐analysis
Source: Mol Genet Genomic Med. 2019 Feb 27;7(5):e611. doi: 10.1002/mgg3.611 (PMC6503061; doi:10.1002/mgg3.611)
Supplement: Supplementary file 1 [file MGG3-7-e611-s001.docx]

**Forest plots of investigated polymorphisms**


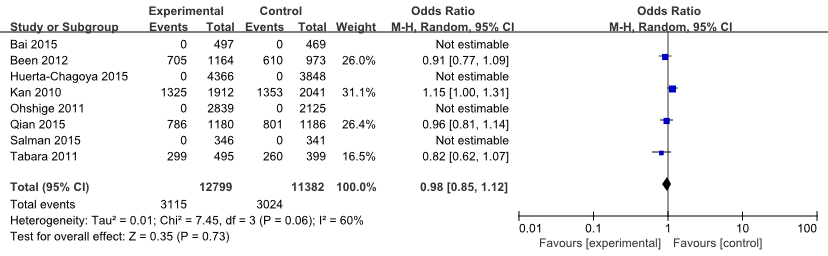


Forest plot of the **rs1387153** polymorphism and T2DM under dominant comparison.


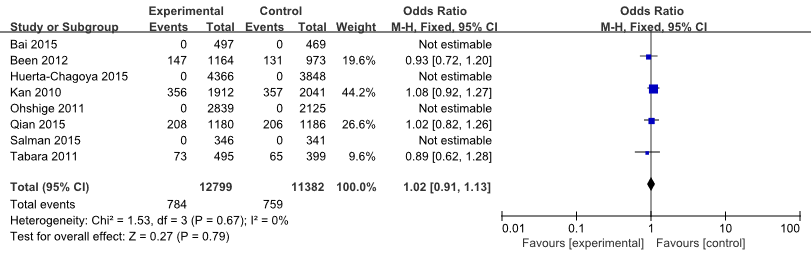


Forest plot of the **rs1387153** polymorphism and T2DM under recessive comparison.


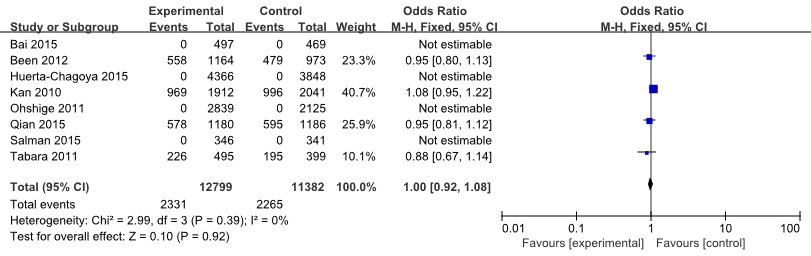


Forest plot of the **rs1387153** polymorphism and T2DM under additive comparison.


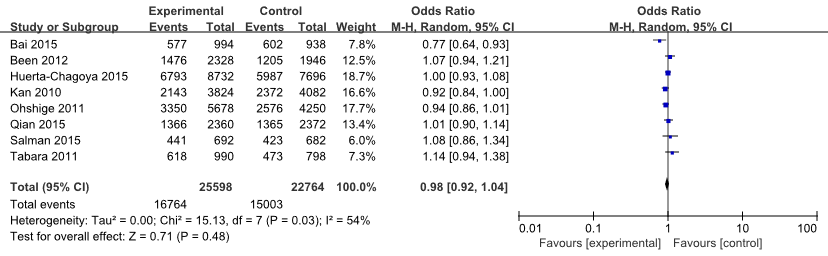


Forest plot of the **rs1387153** polymorphism and T2DM under allele comparison.


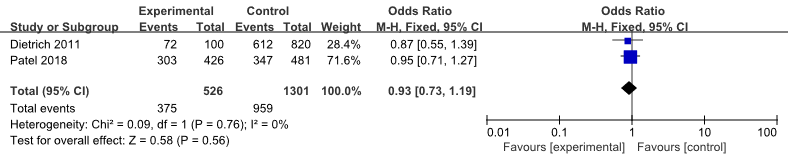


Forest plot of the **rs4753426** polymorphism and T2DM under dominant comparison.


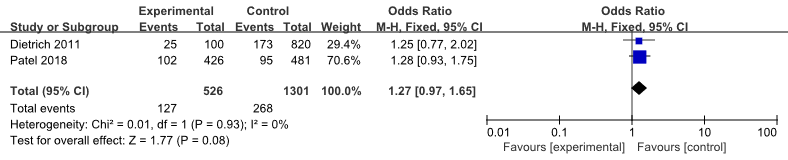


Forest plot of the **rs4753426** polymorphism and T2DM under recessive comparison.


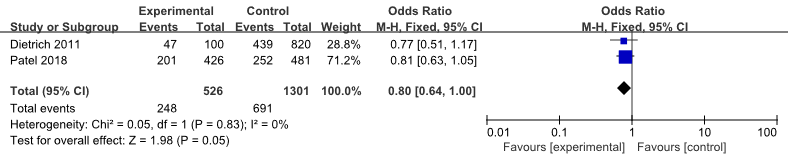


Forest plot of the **rs4753426** polymorphism and T2DM under additive comparison.


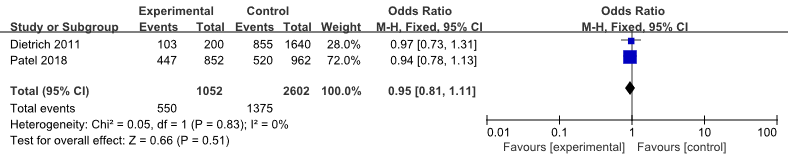


Forest plot of the **rs4753426** polymorphism and T2DM under allele comparison.


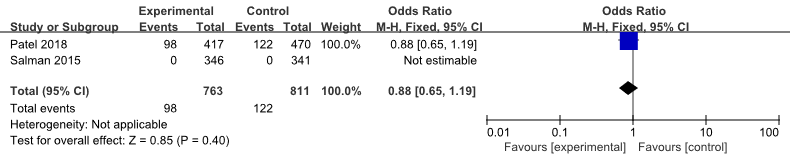


Forest plot of the **rs10830962** polymorphism and T2DM under dominant comparison.


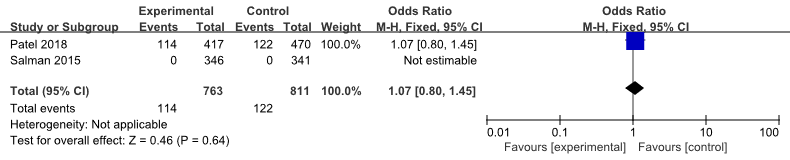


Forest plot of the **rs10830962** polymorphism and T2DM under recessive comparison.


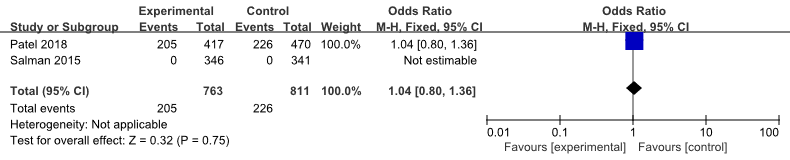


Forest plot of the **rs10830962** polymorphism and T2DM under additive comparison.


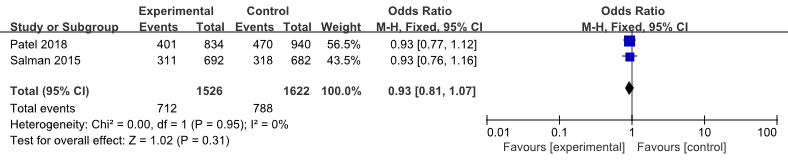


Forest plot of the **rs10830962** polymorphism and T2DM under allele comparison.


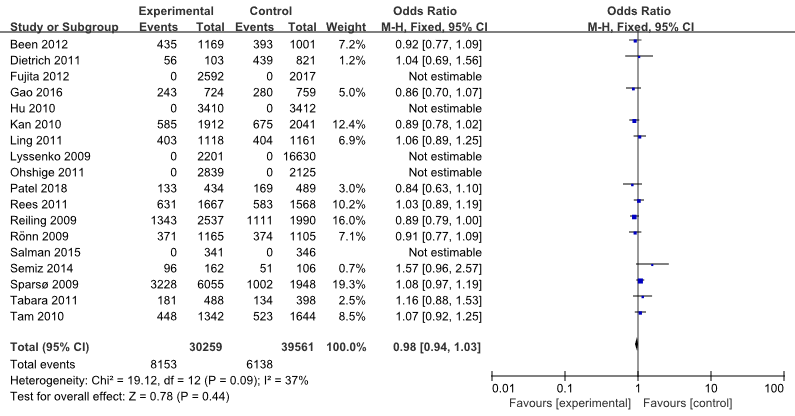


Forest plot of the **rs10830963** polymorphism and T2DM under dominant comparison.


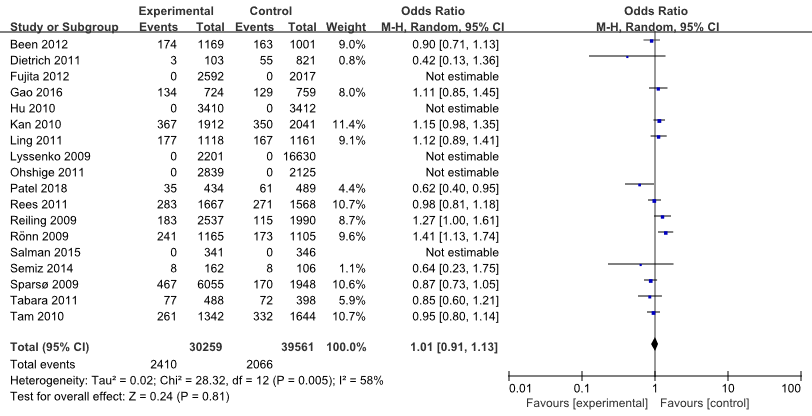


Forest plot of the **rs10830963** polymorphism and T2DM under recessive comparison.


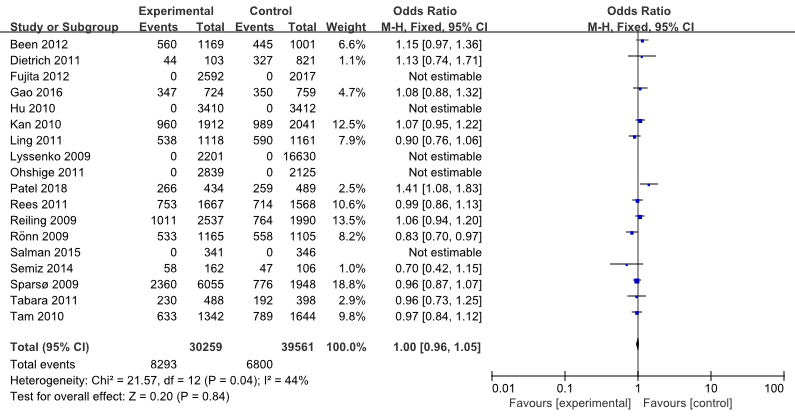


Forest plot of the **rs10830963** polymorphism and T2DM under additive comparison.


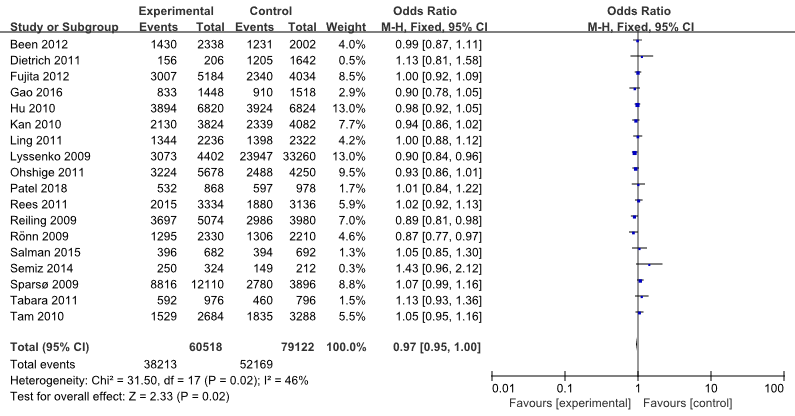


Forest plot of the **rs10830963** polymorphism and T2DM under allele comparison.
